# Immune Responses and Protection Profiles in Mice Induced by Subunit Vaccine Candidates Based on the Extracellular Domain Antigen of Respiratory Syncytial Virus G Protein Combined with Different Adjuvants

**DOI:** 10.3390/vaccines12060686

**Published:** 2024-06-19

**Authors:** Ruiwen Han, Tangqi Wang, Xueting Cheng, Jialuo Bing, Jia Li, Yao Deng, Xuchang Shan, Xuejie Zhang, Donghong Wang, Shucai Sun, Wenjie Tan

**Affiliations:** 1 Zhejiang Provincial Key Laboratory of Medical Genetics, School of Laboratory Medicine and Life Sciences, Wenzhou Medical University, Wenzhou 325035, China; 18267839995@163.com (R.H.); wangtangqi00@163.com (T.W.); ljiawmu@123.com (J.L.); 2 National Key Laboratory of Intelligent Tracking and Forecasting for Infectious Diseases, Key Laboratory of Biosafety, National Health Commissions, National Institute for Viral Disease Control and Prevention, China CDC, 155 Changbai Road, Beijing 102206, China; chengxuetingabc@163.com (X.C.); dengyao@ivdc.chinacdc.cn (Y.D.);; 3 School of Public Health, Xinxiang Medical University, Xinxiang 453003, China; 18755864920@163.com (J.B.); 13675525532@163.com (X.S.); zxj_ggws@163.com (X.Z.); 4 Department of Nuclear Medicine, The Second Hospital of Hebei Medical University, Shijiazhuang 050004, China; 13933189361@163.com

**Keywords:** respiratory syncytial virus, adjuvant, G protein, vaccine, humoral immunity, cellular immunity

## Abstract

Respiratory syncytial virus (RSV) is a leading cause of severe lower respiratory tract disease of infants and older people. There is an urgent need for safe and effective vaccines against RSV infection. In this study, we analyzed the effects of the immune response and protection with the RSV recombinant G protein extracellular domain (G^ecto^) combined with various adjuvants as novel subunit vaccines in mice. All groups receiving RSV G^ecto^ combined with adjuvants exhibited robust humoral and cellular immunity compared to those receiving an adjuvant alone or inactivated RSV vaccine. The greatest effect was observed in mice receiving G^ecto^ combined with a CpG ODN + Alum salt adjuvant, resulting in the highest production of neutralizing antibodies against both RSV A and B subtypes, G-specific IgG and IFN-γ production in splenocytes, and interleukin-2 and interferon-γ expression in CD4^+^ T cells. Significant humoral and cellular immune responses were observed in mice immunized with G^ecto^ combined with AddaS03™ or cyclosporin A adjuvants. The vaccine containing the AddaS03™ adjuvant showed significantly high expression of interleukin-4 in CD4^+^ T cells. Cross-protection against a challenge with either RSV A or B subtypes was observed in the G^ecto^ plus adjuvant groups, resulting in a significant decrease in viral load and reduced pathological damage in the mouse lungs. These findings offer valuable insights into the development and application of recombinant RSV G-subunit vaccines with adjuvants.

## 1. Introduction

Respiratory syncytial virus (RSV) infection causes significant morbidity and mortality in infants, patients who are immunocompromised, and older individuals [1]. RSV belongs to the Pneumoviridae family and *Orthopneumovirus* genus, and is an enveloped, non-segmented, single-stranded negative-sense RNA virus. Its genome is 15.2 kb and contains 10 genes that encode 11 proteins. RSV has a single serotype that is divided into two subtypes, A and B, which alternate their global circulation patterns [2]. RSV is highly contagious and causes acute respiratory tract infections in individuals of all ages. Its transmission is seasonal, typically starting in autumn and peaking during winter. In 2019, there were approximately 3.5 million cases of acute lower respiratory tract infections caused by RSV in children aged below 5 years in China, accounting for over 10% of the global cases. In the older population, RSV is a common cause of lower respiratory tract diseases that can lead to life-threatening pneumonia and bronchitis. Therefore, effective antiviral agents and vaccines for high-risk individuals are urgently needed.

RSV contains two immunogenic envelope glycoproteins, the fusion (F) protein and attachment glycoprotein (G) [3], which are capable of inducing neutralizing antibodies (nAbs) and are the preferred targets for vaccine development. RSV G is produced in infected cells as a full-length, membrane-bound form that is responsible for viral attachment [4] or as a secreted isoform that is involved in immune evasion [5]. RSV G contains a highly conserved non-glycosylated region, known as the central conserved domain (CCD), which interacts with CX3CR1 [6,7], facilitating viral attachment to human respiratory epithelial cells and serving as a target for broad nAbs [8,9,10]. RSV G plays a crucial role in RSV infections and host immune damage. Animal studies have shown that antibodies targeting RSV G reduce the viral load and disease severity [11,12,13], demonstrating that they can modulate host responses to inflammation and disease severity. 

Immunization with the inactivated RSV vaccine FI-RSV results in abnormal immune responses to natural infections and leads to the development of vaccine-enhanced disease (VED) [14,15]. To date, many vaccine candidates are under clinical development using six different approaches—live attenuated, recombinant vector, subunit, particle-based, chimeric, and nucleic acid-based vaccines. Most ongoing efforts in RSV vaccine development have focused on the F protein, and two subunit vaccines have been approved by the USA Food and Drug Administration, which primarily induce effective nAbs through the prefusion conformation of RSV F. Both vaccines were authorized for preventing lower respiratory tract diseases caused by RSV in individuals aged ≥60 years. Despite these advancements, gaps remain in the field of RSV prevention, and the development of RSV vaccines continues to face numerous challenges. Several studies have demonstrated the key role of human T cells in controlling RSV infection, supporting the development of RSV vaccines that also elicit effective T cell responses to improve the efficacy of RSV vaccines. The immune protection induced by RSV G has important clinical significance. Previous studies have demonstrated that RSV G S177Q, a protein single-point mutant, exhibits enhanced immunogenicity and safety compared to the wild-type RSV G while retaining a conformational epitope with high affinity for protective antibodies, making it a promising candidate for an RSV vaccine immunogen [16,17].

The application and optimization of adjuvants may be particularly important for vaccinations with subunit protein antigens, which often fail to elicit sufficient protective immune responses [18,19]. In addition to the commonly used aluminum adjuvants, previous studies have reported several novel adjuvants that can simultaneously enhance nAb and the cellular immune responses induced by subunit vaccines. CpG oligodeoxynucleotides (ODNs) are short, synthetic, single-stranded DNA molecules containing non-methylated CpG dinucleotide motifs that can increase the antibody levels and induce strong Th1-type cellular immune responses [20,21,22]. CpG ODN 1018 was initially approved for use in the hepatitis B vaccine. Superior immunity and protection results were exhibited when subunit vaccine candidates were co-administered with CpG ODN and an alum adjuvant [23]. AddaS03™ is a water-in-oil nanoemulsion adjuvant with a formulation highly similar to the AS03^®^ adjuvant system, which has been successfully used in influenza vaccines [24,25]. Immunization with recombinant RSV G produced in *Escherichia coli* combined with cyclosporine A (CsA) enhanced anti-G nAbs and Treg cells in mice, resulting in protection against RSV challenge without VED [26]. However, no comparative studies have reported the immune response and protection profiles in mice induced by the RSV subunit vaccine in combination with these adjuvants [27,28,29,30,31]. The G protein is a potentially effective vaccine antigen candidate; however, G-based vaccines are also associated with VED in mice, including eosinophilic lung inflammation following RSV challenge. Therefore, there is an urgent need to develop a novel G-based vaccine with enhanced safety and efficacy in combination with optimized adjuvants.

In this study, we compared the effects of different adjuvants, CpG + Al, AddaS03™, and CsA, in combination with the RSV G^ecto^ protein, on immune responses and protection in Balb/c mice. Specifically, we focused on the G-specific antibodies, their subtypes, and nAbs. We also assessed the cellular immune responses and cytokine production. In addition, we evaluated their protective efficacy against challenges with RSV A2 and B9320. Our findings provide valuable information for the development of adjuvant-assisted RSV G-subunit vaccines.

## 2. Materials and Methods

### 2.1. Plasmids, Cells, Viruses, and Animals

**Figure 1:**
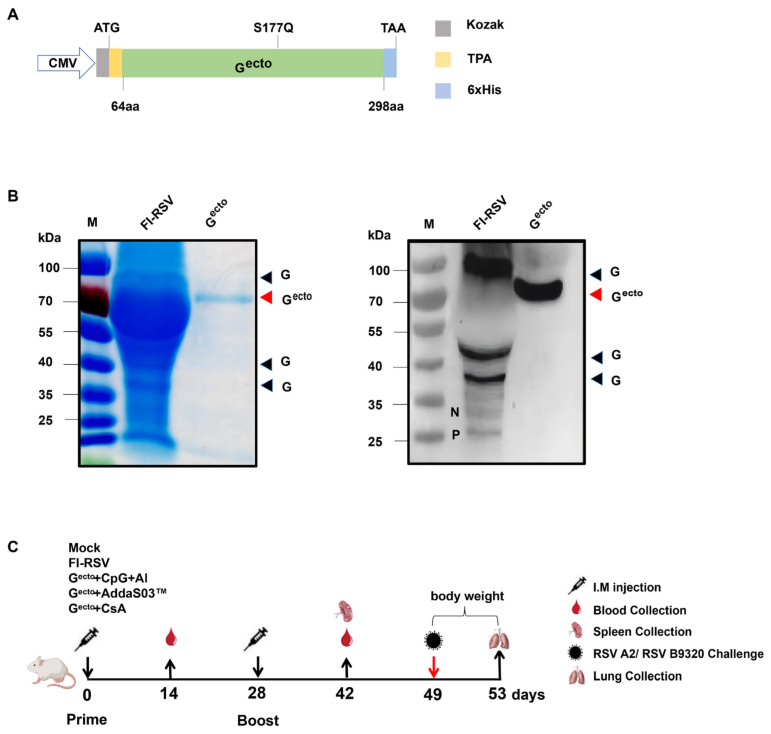
Characterization of the vaccine and scheme of animal experiment in this study: (**A**) construction diagram of the recombinant plasmid pVRC-RSVG^ecto^; (**B**) sodium dodecyl sulfate–polyacrylamide gel electrophoresis and Western blot analyses of purified G^ecto^ protein and G antigen expression in FI-RSV, where the black arrow points to the G protein (varying glycosylation differences), while the red arrow indicates G^ecto^; (**C**) scheme of vaccination, detection, and viral challenge in mice.

The synthetic gene encoding RSV G^ecto^ (64–298 aa, S177Q) from UniProtKB Entry P03423 (Nanjing Kingsley Co., Ltd., Nanjing, China) was cloned into the pVRC vector, together with an N-terminal tissue plasminogen activator and a C-terminal hexahistidine purification tag, to construct the pVRC-RSV G^ecto^ (64–298 aa, S177Q)-His plasmid (Figure 1A). The RSV G^ecto^ S177 mutant was designed based on the known structure of the RSV G that binds to human antibodies. Serine 177 was mutated to glutamine (S177Q) to disrupt its interaction with the CX3C chemokine receptor, CX3CR1 [32]. 

Mycoplasma-free human embryonic kidney epithelial (HEK-293T) African green monkey kidney (Vero) cells were maintained in Dulbecco’s minimal essential medium (Gibco™, Waltham, MA, USA) supplemented with 10% fetal bovine serum (PAN, Cat:P30-3302) and 1% penicillin–streptomycin (PS, Cat:15140-122). The RSV strains A2 and B9320 (ATCC, Manassas, VA, USA) were generously provided by Nanjing Novogene Co., Ltd. (Nanjing, China). The clinical strain hRSV/C-Tan/BJ 202301 was isolated and preserved by the Emergency Technology Center of the Institute for Viral Disease Control and Prevention, Chinese Center for Disease Control and Prevention. Female Balb/c mice aged 6–8 weeks were purchased from Beijing Sibeifu Biotechnology Co., Ltd. (Beijing, China). The vaccination, housing, and viral challenge experiments were conducted at the Animal Center of Beijing Kexing Vaccine Co., Ltd. (Beijing, China), following the guidelines of the Institutional Laboratory Animal Care and Use Committee (Ethics Review Number 20231128092). All invasive mouse experiments were performed under anesthesia.

### 2.2. Vaccine and Adjuvants

The pVRC-RSV G^ecto^ (64–298 aa, S177Q)-His plasmid was transfected into 293T cells using polyethylenimine (PEI). After transfection, the supernatant was collected after 72 h of incubation at 37 °C with 5% CO_2_. The proteins were purified using the ÄKTA pure protein purification system (Cytiva, Marlborough, MA, USA) with a HisTrap Excel affinity column, followed by concentration and buffer exchange in phosphate-buffered saline (PBS). The purity was assessed using Coomassie brilliant blue staining (P1300; Beijing Solarbio Science and Technology Co., Ltd., Beijing, China).

The FI-RSV vaccine was prepared according to the method described by Kim et al. [14]. Briefly, 100 mL of purified RSV was incubated with formalin (37–40%) at a ratio of 4000:1 (*v*/*v*) at 37 °C for 72 h, followed by centrifugation at 50,000× *g* for 1 h at 4 °C. The pellets were diluted in 4 mL of minimal essential medium (MEM), then mixed with an Al(OH)_3_ (10 mg/mL) adjuvant at a ratio of 5:2 (*v*/*v*) and suspended in 1 mL of serum-free MEM for storage at 4 °C.

The aluminum adjuvant was an opalescent liquid with a working concentration of 10 mg/mL. The CpG1826 adjuvant was synthesized by TaKaRa Bio (Kusatsu, Shiga, Japan) and dissolved in physiological saline for use at a dosage of 30 μg CpG1826 + 50 μg Al(OH)_3_ per mouse. The AddaS03™ adjuvant (Beijing Dakowei Biotechnology Co., Ltd., Beijing, China) was a water-in-oil nanoemulsion adjuvant, which mainly consisted of biodegradable squalene, DL-α-tocopherol, and Tween^®^ 80 surfactant, which was administered at a dosage of 50 μL per mouse. CsA (Sigma-Aldrich, St. Louis, MO, USA) is an immunosuppressive agent widely used in organ transplantation and the treatment of pediatric aplastic anemia and other autoimmune diseases. In this study, CsA was used as the adjuvant at a dosage of 10 μg per mouse.

### 2.3. Western Blotting

The purified RSV G^ecto^ protein and FI-RSV were subjected to sodium dodecyl sulfate–polyacrylamide gel electrophoresis at 80 V for 2 h before transfer onto nitrocellulose membranes. After blocking with 5% skim milk, a polyclonal mouse anti-RSV G protein antibody (Ab20745; Abcam, Cambridge, UK) was used as the primary antibody, followed by horseradish peroxidase (HRP)-conjugated goat anti-mouse IgG secondary antibody (ZB-2305; Beijing Zhongshan Golden Bridge Biotechnology Co., Ltd., Beijing, China). The results were imaged using a chemiluminescent imager (Figure 1B).

### 2.4. Animal Experiments 

Seventy-five female Balb/c mice aged 6–8 weeks were divided into five groups, with 15 mice in each group, namely the adjuvant only (Mock), FI-RSV, 10 μg G^ecto^ + adjuvant A (30 μg CpG + 50 μg Al; RSV G^ecto^ + CpG + Al), 10 μg G^ecto^ + adjuvant B (50 μL AddaS03™, *v*/*v* = 1:1; RSV G^ecto^ + AddaS03™), and 10 μg G^ecto^ + adjuvant C (10 μg CsA; RSV G^ecto^ + CsA). The mice were immunized by intramuscular injection (Figure 1C) and received a booster immunization after a 4-week interval. Blood samples were collected from each mouse 14 days after the initial and booster immunizations. The mice were sacrificed via cervical dislocation. Single-cell suspensions were taken from the spleens with a pestle and filter. 

Three weeks after the booster immunization, the mice were intranasally challenged with live RSV A2 or B9320 viruses (2 × 10^6^ PFU/mouse), and their body weights were measured daily. After 4 days of infection, the mice were euthanized and partial lung tissues were collected to measure the RSV load using real-time RT-PCR and 50% tissue culture infectious dose (TCID_50_) assays. Partial lung tissue samples were also used for the histopathological examination. Tissue sections (4 microns) were prepared and stained with hematoxylin–eosin (Beijing Zhongke Wanbang Biotechnology Co., Ltd., Beijing, China) following international standardized diagnostic criteria for pathological evaluations. 

### 2.5. Enzyme-Linked Immunosorbent Assay (ELISA)

A 96-well plate was coated with RSV G antigen (Ga) at 100 ng/well and incubated overnight at 4 °C. After blocking with 10% goat serum at 37 °C for 2 h, three-fold serially diluted sera were plated into each well and incubated at 37 °C for 1 h. Subsequently, the HRP-conjugated goat anti-mouse IgG secondary antibody (ZB-2305; Beijing Zhongshan Golden Bridge Biotechnology Co., Ltd., Beijing, China) (1:5000) was added and incubated at 37 °C for 1 h. The plates were developed with tetramethylbenzidine (TMB) followed by the addition of 2 M H_2_SO_4_ to stop the reaction and read at 450 nm.

For antigen-specific IgG antibody subtype detection, after blocking, the mouse sera were three-fold serially diluted and incubated at 37 °C for 1 h. Subsequently, the HRP-conjugated goat anti-mouse IgG1 antibody (ab97240; Abcam) (1:5000) and HRP-conjugated goat anti-mouse IgG2a antibody (ab97245; Abcam) (1:5000) were added and incubated at 37 °C for 1 h. Color development was performed using the TMB substrate and the reaction was stopped with 2M H_2_SO_4_. The absorbance was measured at 450 nm using an ELISA reader. A sample with an absorbance value greater than or equal to 2.1 times the value of the blank well was considered positive. The highest dilution ratio determined as positive was considered the endpoint, and the reciprocal of the endpoint dilution represented the antibody titer of the sample.

### 2.6. Neutralization Assay 

In a 96-well plate, two-fold serially diluted (50 µL per well) immune sera were combined with 50 µL 10^4^ TCID_50_/mL of RSV A2, B9320, or hRSV/C-Tan/BJ 202301 at a 1:1 ratio and thoroughly mixed. The plate was then incubated at 37 °C with 5% CO_2_. After 1.5 h, 2 × 10^4^ Vero cells were added to the wells at a 1:1 volume ratio. The plate was incubated at 37 °C for a further 6 days until the positive control (virus only) wells showed an 80% cytopathic effect (CPE). The cells were fixed and stained with 0.25% crystal violet in 4% paraformaldehyde. The stained plates were air-dried and evaluated for CPE using a dissecting microscope. The lowest dilution that resulted in 80% CPE inhibition was identified as the endpoint neutralizing antibody titer for that sample. The LOD was assigned as 3. Any sample with a titer less than the LOD was assigned a value of 1 [33]. 

### 2.7. Enzyme-Linked Immunospot Assay (ELISPOT)

Two weeks after the booster immunization, the splenocytes of the mice were collected for an IFN-γ ELISPOT assay. Purified anti-mouse IFN-γ was diluted 1:200 with 1× PBS and used to coat a 96-well ELISPOT plate at 100 μL per well, which was then incubated overnight at 4 °C (BD ELISPOT Set, BD Biosciences, Franklin Lakes, NJ, USA). Subsequently, single-cell suspensions of splenocytes were prepared and a dominant peptide of the G protein consisting of a 20-amino acid sequence (NPTCWAICKRIPNKKPGKKT) was used as the stimulant for the IFN-γ ELISPOT to measure the cellular immune response [34]. This H2-d dominant peptide was selected because it represents both Ga and Gb epitopes, being situated in the central conserved region of the RSV G protein. The spots were scanned and quantified using an ImmunoSpot CTL reader. The spot-forming units (SFUs) per million cells were calculated by subtracting the number of negative control wells. The number of spots was plotted as the mean ± SEM (standard error of the mean) using GraphPad Prism 9.0 software.

### 2.8. Intracellular Cytokine Staining (ICS)

Splenic tissues obtained from the mice 2 weeks after the booster immunization were prepared as single-cell suspensions at a concentration of 2 × 10^6^ cells per tube. Five micrograms of the RSV G-specific stimulation peptide (NPTCWAICKRIPNKKPGKKT) was added to each tube and incubated at 37 °C with 5% CO_2_ for 30 min. A stimulation blocker (555029; BD Biosciences) was added and mixed thoroughly. The cell culture was placed in a 37 °C CO_2_ incubator for less than 12 h and then centrifuged at 350× *g* for 5 min. The cell pellets were resuspended in 1× DPBS, then 0.6 μL of FVS780 dye (565388; BD Biosciences) was added and gently vortexed. The mixture was then incubated at room temperature in the dark for 15 min. Two milliliters of 1% fetal bovine serum in PBS was added to the flow cytometry tube before centrifuging at 350× *g* for 5 min and discarding the supernatant. Next, 2 μL of Fc Block antibody (553141; BD Biosciences) was added to each tube and incubated on ice for 5–10 min to block Fc receptors. Surface staining was performed by adding CD3 (551163; BD), CD8 (553030; BD), and CD4 (563106; BD Biosciences) antibodies to the tubes and incubating them at 4 °C in the dark for 15 min. Subsequently, 1 mL of cell-staining buffer (554656; BD Biosciences) was added. The cell pellet was resuspended in 250 μL of Fixation/Permeabilization Solution (565388; BD Biosciences) and incubated at 4 °C in the dark for 20 min. Intracellular staining was performed by incubating the cells with IL-2 (554428; BD Biosciences), IL-4 (554436; BD Bioscience), TNF (557644; BD Bioscience), and IFN-γ (563376; BD Bioscience) antibodies at 4 °C for 30 min. The data were collected using FACS II and analyzed using FlowJo software (version 10.8.1).

### 2.9. Real-Time RT PCR

The lung tissue was homogenized using a tissue grinder to obtain a uniform suspension. After centrifugation at 1000× *g* for 10 min, the supernatant was collected. A total of 150 μL was then used for RNA extraction using the nucleic acid extraction kit, according to the manufacturer’s protocol (Nanjing Novogene Co., Ltd., RM501-02). The following primer and probe sequences were used for the RSV A gene: upstream primer sequence: 5′-AGATCAACTTCTGTCATCCAGCAA-3′; downstream primer sequence: 5′-GCACATCATAATTAGGAGCATCAAT-3′; probe sequence: 5′-(FAM) CACATCCAACGGAGCACAGGAGAT (TAMRA)-3′. For the RSV B gene, the primer and probe sequences were as follows: upstream primer sequence: 5′-AAGATGCAAATCATAAATTCACAGGA-3′; downstream primer sequence: 5′-TGATATCCAGCATATTTAAGTATCTTTATAGTG-3′; probe sequence: 5′-(FAM)FAM-AGGTATGTTATATGCTATGTCCAGGTTAGGAAGGGAA (TAMRA)-3′. The amplifications were performed using the One-Step PrimeScript™ RT-PCR Kit (Perfect Real Time), according to the manufacturer’s instructions (RR064A; TaKaRa Bio, Kusatsu, Japan).

### 2.10. Statistical Analyses

Two-group comparisons were tested, depending on whether the data were normally distributed, using a Student’s *t* test or Mann–Whitney test (unpaired). An unpaired Student’s *t* test and ANOVA were used in one-way or two-way data analyses. A linear mixed-effects model was used to assess the endpoint for each group, with the timepoint as the fixed effect and participant as the random effect. The data are presented as the mean ± SEM (ns: *p* > 0.05; *: *p* < 0.05; **: *p* < 0.01; ***: *p* < 0.001). GraphPad Prism version 9.0 (GraphPad Software, Boston, MA, USA) was used for data processing, statistical analyses, and graph generation.

## 3. Results

### 3.1. Expression and Purification of RSV G^ecto^ Protein

The recombinant RSV G^ecto^ was expressed and secreted in 293T cells. After purification via affinity chromatography, the purity and expression of the RSV G^ecto^ protein were confirmed using Coomassie brilliant blue staining and a Western blot analysis, respectively, validating its location and expression in FI-RSV (Figure 1B). The black arrows in Figure 1B denote glycosylation differences in the full-length G protein, while G^ecto^ is represented by a single band due to its truncation at the extracellular region, consistent with previous findings [17,35]. 

### 3.2. Humoral Immune Response Profiles in Mice Induced by RSV G^ecto^ Combined with Different Adjuvants

**Figure 2:**
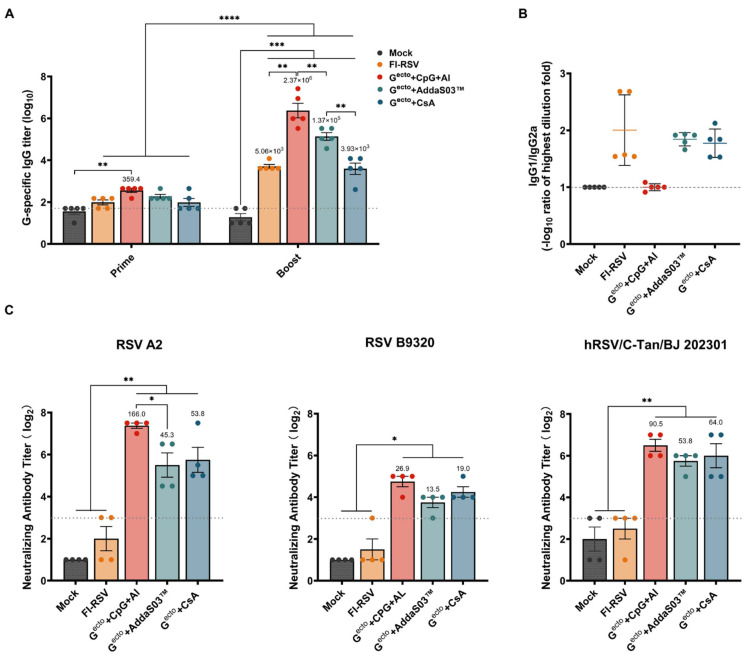
Humoral immune response profiles in Balb/c mice induced by RSV G^ecto^ with different adjuvants and RSV-FI vaccines. (**A**) Detection of G-specific serum IgG antibodies (n = 5); a linear mixed-effects model approach was adopted here to analyze the data on antibody titers (prime and boost) between the control and immunized groups. (**B**) Ratio of G-specific serum IgG1/IgG2a antibodies at 14 days after the booster immunization (n = 5). (**C**) Detection of serum-neutralizing antibodies (nAbs) against RSV A2, RSV B9320, and hRSV/C-Tan/BJ 202301 at 14 days after the booster immunization (n = 4). Statistical significance was analyzed using a one-way analysis of variance. The bars plotted show means ± SEM. The results represent three independent experiments (* *p* < 0.05, ** *p* < 0.01, *** *p* < 0.001, **** *p* < 0.0001).

The immunization procedure for the mice is illustrated in Figure 1C. Two weeks after the initial and booster immunizations, the serum humoral immune response profiles in mice were assessed (Figure 2). After the booster immunization, higher levels of RSV G^ecto^-specific antibodies were observed compared to the initial immunization. The RSV G^ecto^ + CpG + Al and RSV G^ecto^ + AddaS03™ groups showed significantly higher titers of specific antibodies (2.37 × 10^6^ and 1.37 × 10^5^, respectively) than did the RSV G^ecto^ + CsA (4 × 10^3^) and FI-RSV (5.06 × 10^3^) groups (*p* < 0.01) (Figure 2A).

The analysis of the antibody subtypes IgG1 and IgG2a (Figure 2B) revealed that except for the RSV G^ecto^ + CpG + Al group, the titers of IgG1 were significantly higher than IgG2a after the booster immunization with either RSV G^ecto^ + AddaS03™ or RSV G^ecto^ + CsA, indicating that the vaccine did not induce a preferential Th1-type antibody immune response (Figure 2B). The immunization with RSV G^ecto^ + CpG + Al elicited a strong and balanced IgG1 and IgG2a response, demonstrating a balanced immune response (Figure 2B).

The results of the neutralization assay for the serum samples collected 14 days after the second immunization showed that all RSV G^ecto^-combined adjuvant groups induced the production of significant nAbs against both RSV A2 and B9320 (Figure 2C) compared to both the FI-RSV and mock groups (*p* < 0.05). Similar results were achieved using the epidemic strains isolated from Chinese patients (hRSV/C-Tan/BJ 202301, belonging to the ON1 subgroup of subtype A). 

### 3.3. Cellular Immunity Profiles of Mice Induced by RSV G^ecto^ Combined with Different Adjuvants

**Figure 3:**
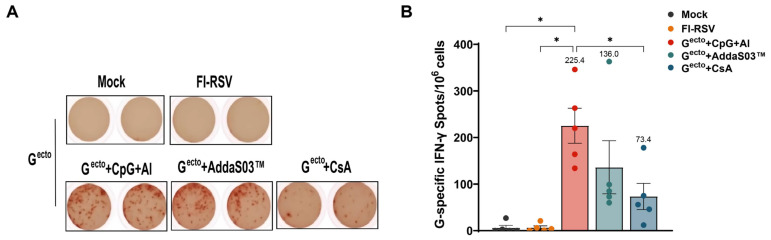
Assessment of the RSV G-specific cellular immune response in Balb/c mice using the ELISPOT assay: (**A**,**B**) representative plots for IFN-γ expression. Splenocytes were stimulated with RSV G-specific stimulation peptide (NPTCWAICKRIPNKKPGKKT) and ELISPOT assay results for RSV G-specific IFN-γ-secreting spots per million splenocyte mononuclear cells (n = 5). The spots in **B** represent detection result from five mice each group (* *p* < 0.05).

**Figure 4:**
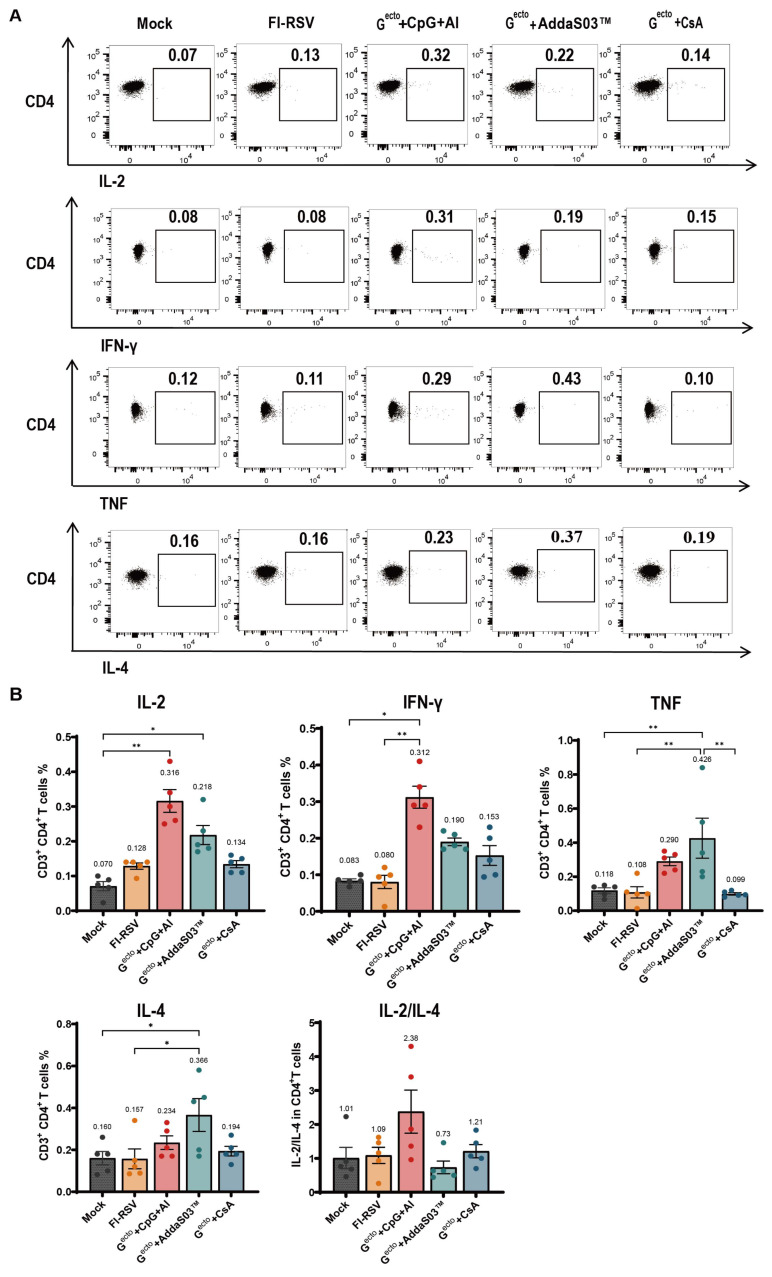
Detection of intracellular cytokines IFN-γ, TNF, IL-2, and IL-4 in CD4^+^ T cells from G^ecto^-immunized mice. (**A**) Representative flow cytometry plots for intracellular cytokines expression by CD4^+^ T cells. Splenocytes were stimulated with the RSV G-specific stimulation peptide, which was assessed via a flow cytometry assay (n = 5). (**B**) Percentages of RSV G-specific cytokine-positive cells among total CD4^+^ T cell populations in the splenocytes of mice detected by ICS (n = 5). The spots in (**B**) represent detection result from five mice each group (* *p* < 0.05, ** *p* < 0.01).

The cellular immunity in the mice 2 weeks after the booster immunization was assessed using the ELISPOT assay and ICS. The ELISPOT assay showed that the RSV G^ecto^ + CpG + Al, RSV G^ecto^ + AddaS03™, and RSV G^ecto^ + CsA groups induced a significantly higher level than the mock and FI-RSV groups of G-specific cellular immunity, as shown by spot-forming cell counts of 225/10^6^, 136/10^6^, and 73/10^6^ SMNCs, respectively (Figure 3). No G-specific cellular immunity was observed in the FI-RSV or control groups. In addition, the ICS showed the secretion of multiple cytokines in CD4^+^ T cells of the splenocytes from mice immunized with RSV G^ecto^ (Figure 4A,B and the Supplementary Materials). The RSV G^ecto^ + CpG + Al group exhibited significantly higher expression rates of IL-2 (0.316%) and IFN-γ (0.312%) in CD4^+^ T cells than did the other groups, with the RSV G^ecto^ + AddaS03™ group showing the next highest expression rate. The RSV G^ecto^ + AddaS03™ group showed significantly higher expression rates of TNF-α (0.426%) and IL-4 (0.366%) in CD4^+^ T cells than did the control group. 

No significant cytokine secretion in the CD8^+^ T cells of immunized mice was detected using ICS (the Supplementary Materials).

### 3.4. Protective Efficacy against RSV A2 and B9320 in Mice Induced by RSV G^ecto^ Combined with Different Adjuvants

**Figure 5:**
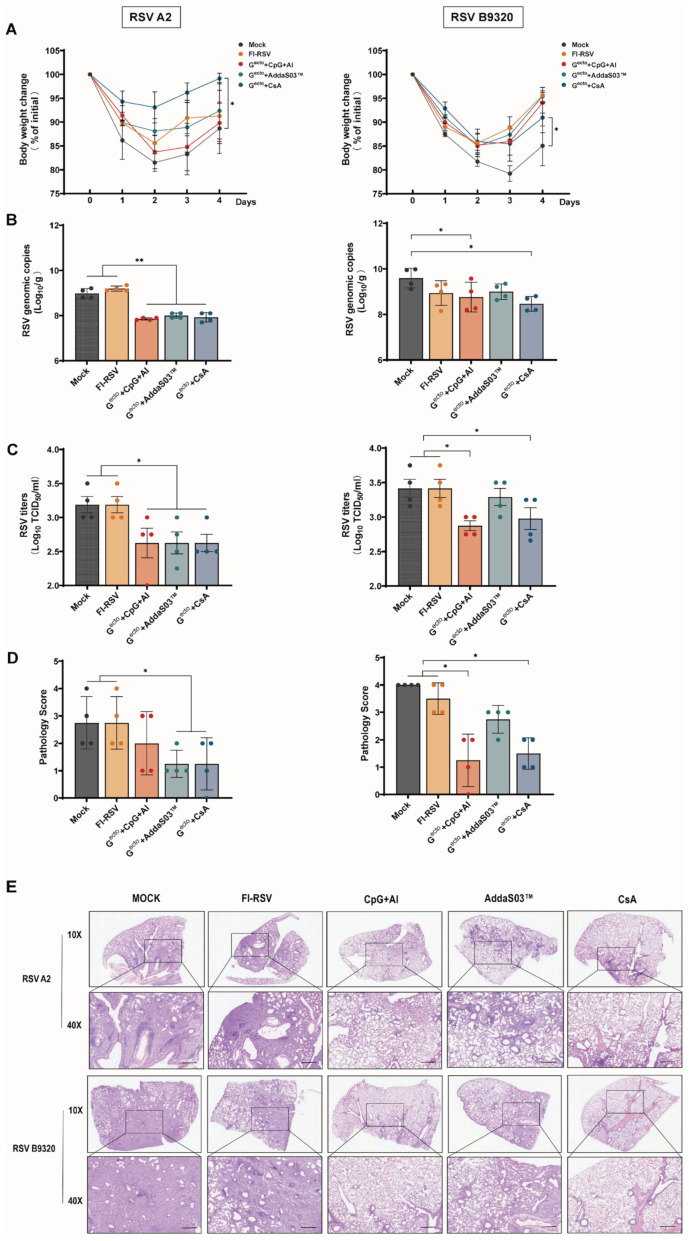
Immune protection experiment for the RSV G^ecto^ subunit vaccine in mice challenged with RSV. (**A**) Monitoring of body weight after RSV A2 and RSV B9320 challenges (n = 5); a linear mixed-effects model approach was adopted here to analyze the data between the control and immunized groups. (**B**) Lung viral load detection using real-time RT-PCR at 4 days after RSV A2 or B9320 challenge (n = 4). (**C**) Lung viral titers determined by TCID50 assay at 4 days after RSV A2 or B9320 challenge (n = 4). (**D**) International Harmonisation of Nomenclature and Diagnostic Criteria (INHAND) scores of challenged mice lungs at 4 days after RSV A2 or B9320 challenge, on a severity scale of 0–3 (none, mild, moderate, and severe). Statistical significance for groups (n = 4/group) of a one-way ANOVA is shown (* *p* < 0.05). (**E**) The pulmonary histopathological analysis at 4 days after RSV A2 or B9320 challenge. Scale bar = 200 µm.

The results of the viral challenge experiment showed that in all groups, the mice reached their lowest body weights on day 2 after RSV A2 challenge or on day 3 after RSV B9320 challenge, followed by gradual recovery (Figure 5A). Compared to the RSV G^ecto^-immunized groups, the mock group had higher rates of weight loss than all the G^ecto^-immunized groups.

The RSV load in the lung samples of the mice on day 4 after challenge was assessed using real-time RT-PCR and TCID_50_ assays. The real-time RT-PCR results showed that after the RSV A2 challenge, the RSV G^ecto^ + CpG + Al, RSV G^ecto^ + AddaS03™, and RSV G^ecto^ + CsA groups exhibited reduced numbers of RSV genome copies in the lung tissue compared to the mock and FI-RSV groups (Figure 5B). Similarly, after RSV B9320 challenge, the RSV G^ecto^ + CpG + Al and RSV G^ecto^ + CsA groups showed reduced numbers of RSV genome copies in the lungs compared to the mock and FI-RSV groups. The FI-RSV group did not show a significant difference in lung viral load compared to the mock group after RSV A2 or B9320 challenge. The TCID_50_ assay after RSV A2 or B9320 challenge showed similarly reduced RSV loads in the lungs of mice immunized with RSV G^ecto^ compared to the mock and FI-RSV groups (Figure 5C).

The pathological analysis of the lung tissue from mice was performed 4 days after the RSV challenge. Compared to the control group, all RSV G^ecto^-immunized groups showed reductions in interstitial pneumonia lesions after RSV A2 or B9320 challenge (Figure 5D,E). Both the mock and FI-RSV groups had higher pathological scores than the RSV G^ecto^ immunization groups. The RSV G^ecto^ + CpG + Al and RSV G^ecto^ + CsA groups exhibited milder pathological injury rates than the RSV G^ecto^ + AddaS03™ group, which is consistent with the RSV load reduction detected using the TCID_50_ assay.

## 4. Discussion

Two subunit vaccines targeting the Pre-F protein have been approved for preventing lower respiratory tract disease in older adults [36]. Therefore, there is an urgent need to develop safer and more effective vaccines to prevent RSV infections in children and adults. Further research into the antigen design and optimized adjuvants is crucial, particularly for the pediatric population. In this study, we investigated the immunogenicity and protective effects of the less-explored RSV G^ecto^ protein antigen combined with different adjuvants using a prime–boost immunization strategy in mice. We systematically compared the CpG + Al, AddaS03™, and CSA adjuvants in terms of their antibody production, cellular immunity, and protection. Our results suggest that RSV G^ecto^ combined with these adjuvants elicited high levels of antigen-specific humoral and cellular immunity in mice and provided protection against RSV infection. Our findings underscore the potential of this approach for vaccine development. Future investigations will involve comparisons of the compositions of existing vaccines with other commercially available RSV vaccines. These comparative studies will contribute to a better understanding of vaccine efficacy and guide the development of improved preventive strategies against RSV infections.

The currently available vaccines use the RSV preF protein as an immunogen. However, obtaining a stable prefusion form of the recombinant F protein is technically challenging, and the post-fusion or misfolded forms of the F protein have a weak ability to induce sufficient nAbs. In contrast, the immunogenicity of the G protein is less affected by the protein structure. Increasing evidence suggests the importance of the CCD and CX3C motifs of the G protein in the design of RSV vaccines. For instance, the immunization of mice with RSV G protein or peptides containing the CCD induces protective neutralizing antibody responses and blocks the interaction between the G protein, CX3C, and CX3CR1 [37,38]. This prevents RSV-mediated disease onset, with the CX3C motif being important for inducing protective immune responses [39]. In animal models, antibodies targeting RSV G have been shown to reduce the viral load and disease severity. High concentrations of RSV G antibodies in the serum are correlated with mild RSV disease in infants, and the immune protection induced by RSV G has several significant clinical implications. Therefore, further research on RSV G proteins is important and valuable.

Combining recombinant subunit vaccines with adjuvants is a common immunization strategy aimed at enhancing the immune response and protective efficacy. Previous studies have demonstrated that the G protein, when administered without adjuvants, exhibits limited immunogenicity in mice [40], even falling below the response observed in the FI-RSV group [26]. Consequently, Gecto without adjuvants was excluded from consideration, with FI-RSV serving as the comparator in this study. Furthermore, historical investigations have linked FI-RSV vaccination with the potential for inducing severe vaccine-enhanced disease (VED) [35]. Consequently, FI-RSV is frequently utilized as a control in RSV vaccine studies to validate the protective effects of candidate RSV vaccines.

Initially, the FI-RSV vaccine showed good tolerability in infants; however, vaccinated infants exhibited symptoms of VED, which might primarily be attributed to non-nAbs, low-titer nAbs, or a Th2-cell-based T-cell response induced by the vaccine. In this study, we confirmed similar immune profiles and VED in mice induced by RSV-FI vaccination and post-RSV challenge.

CpG ODN induces a Th1-type immune response, resulting in strong humoral and cellular immunity [20,21,22]. In this study, the CpG + Al adjuvant combined with RSV G^ecto^ significantly enhanced the Th1-type immune response in mice and exhibited significantly higher levels of antigen-specific IgG, nAb, and expression levels of IL-2 and IFN-γin CD4^+^ T cells than the other adjuvants, resulting in better cross-protection against the RSV A and B subtypes than both the RSV G^ecto^ + AddaS03™ and FI-RSV vaccines. 

AS03 is a classic oil-in-water emulsion adjuvant that was approved for use in the influenza vaccines Pandemrix, TM, and GSK during the 2009 H1N1 pandemic in the European Union. In 2013, the Food and Drug Administration approved AS03 for use as an H5N1 avian influenza vaccine. Currently, AS03 is primarily used in various influenza vaccines to enhance antibody titers and improve their persistence [24,25]. In this study, we used the AS03 analog AddaS03™ as the adjuvant for the RSV G^ecto^ vaccine. This treatment elicited robust humoral and cellular immunity and showed significantly higher expression levels of TNF-α and IL-4 in CD4^+^ T cells than the control group. RSV G^ecto^ + AddaS03™ resulted in slight protection against RSV A2 but failed to generate effective cross-protection against RSV B, which may be the result of higher levels of nAbs targeting RSV A2 and lower levels of nAbs targeting RSV B.

Wang et al. [26] reported that in mice, immunization with the RSV G protein combined with CsA induced higher levels of nAbs and significantly increased the Treg cell levels to inhibit lung VED after RSV infection, thereby providing protection against viral challenge. In this study, the RSV G^ecto^ + CsA immunization group also elicited significant levels of nAbs against both RSV A and B subtypes, although the cellular immunity was lower than that of RSV G^ecto^ + CpG + Al. We further confirmed that the RSV G^ecto^ + CsA immunization provided better cross-protection against RSV A2 or B9320 infection, indicating a significantly reduced lung viral load and milder pathological injury to the lung tissue of mice.

The RSV-A and RSV-B strains often co-circulate during outbreaks, emphasizing the importance of developing preventive options that can target both strains. Previous reports suggest that the available monoclonal antibodies (e.g., palivizumab and nirsevimab) and vaccines, even if targeting RSV-A only, demonstrate substantial efficacy against RSV-B. In this study, while cross-neutralization and -protection were observed in mice receiving RSV Gecto (based on RSV-A only) combined with adjuvants, heterogeneous responses to the assessed strains of RSV were also noted. Future studies will investigate whether the differences in efficacy against RSV-B observed in this preclinical research translate into clinical disparities. Moreover, it is essential to explore the potential of combining optimized G antigens from both RSV-A and RSV-B or investigating alternative targets for RSV vaccine development.

This study had some limitations, including the lack of protein or adjuvant dose comparisons during the subunit vaccine immunization, suggesting that the protein or adjuvant combination used may not be optimal. RSV G^ecto^ combined with CpG + Al showed superior immune responses compared to CsA but did not exhibit stronger protective efficacy, and further investigation of the mechanism involved is required. 

In conclusion, we evaluated the immunogenicity and protective efficacy of the less explored RSV G^ecto^ protein antigen combined with various adjuvants using a prime–boost immunization strategy in mice in this study. Our data suggest that the immune responses and protection profiles in mice induced by RSV G^ecto^ may be attributed to adjuvant application. This requires further exploration of the underlying mechanisms and validation of its optimized application using other animal models. 

## Data Availability

The datasets generated during the current study are available from the corresponding author on reasonable request.

## Supplementary Materials

The following supporting information can be downloaded at https://www.mdpi.com/article/10.3390/vaccines12060686/s1: Figure S1. Detection of intracellular cytokines IFN-γ, IL-2, TNF, and IL-4 in CD8^+^ T cells from Gecto-immunized mice. Figure S2. Gating strategy for the detection of CD4^+^ T cells in intracellular cytokines. Exemplary gating strategy of splenocyte samples from a G^ecto^-immunized mouse. Splenocytes were collected 14 days later and the expression of IL-2, IFN-γ, TNF, and IL-4 in CD4^+^ T cells was measured.
