# Supplementary material for: Immune Responses and Protection Profiles in Mice Induced by Subunit Vaccine Candidates Based on the Extracellular Domain Antigen of Respiratory Syncytial Virus G Protein Combined with Different Adjuvants
Source: Vaccines (Basel). 2024 Jun 19;12(6):686. doi: 10.3390/vaccines12060686 (PMC11209252; doi:10.3390/vaccines12060686)
Supplement: Supplementary file 1 [file vaccines-12-00686-s001.zip › vaccines-3004428-supplementary-updating.pdf]

Supplemental files

Immune Responses and Protection Profiles in Mice Induced by Subunit Vaccine Candidates Based on the Extracellular Domain Antigen of Respiratory Syncytial Virus G Protein Combined with Different Adjuvants

Ruiwen Han [1], Tangqi Wang [1†], Xueting Cheng [1], Jialuo Bing [2], Jia Li [3], Yao Deng [1], Xuchang Shan [2], Xuejie Zhang [2], Donghong Wang [1], Shucai Sun [4] and Wenjie Tan [1,2,3,4*]

1 Key Laboratory of Biosafety , National Health Commissions, National Institute for Viral Disease Control and Prevention, China CDC, 155 Changbai Road, Beijing 102206, China; 18267839995@163.com
2 School of Public Health，Xinxiang Medical University，Xinxiang 453003，China
3 Zhejiang Provincial Key Laboratory of Medical Genetics，School of Laboratory Medicine and Life Sciences，Wenzhou Medical University，Wenzhou 325035，China
2 Department of Nuclear Medicine，The Second Hospital of Hebei Medical University，Shijiazhuang 050000，China
* Correspondence: tanwj@ivdc.chinacdc.cn
† These authors contributed equally to this work.

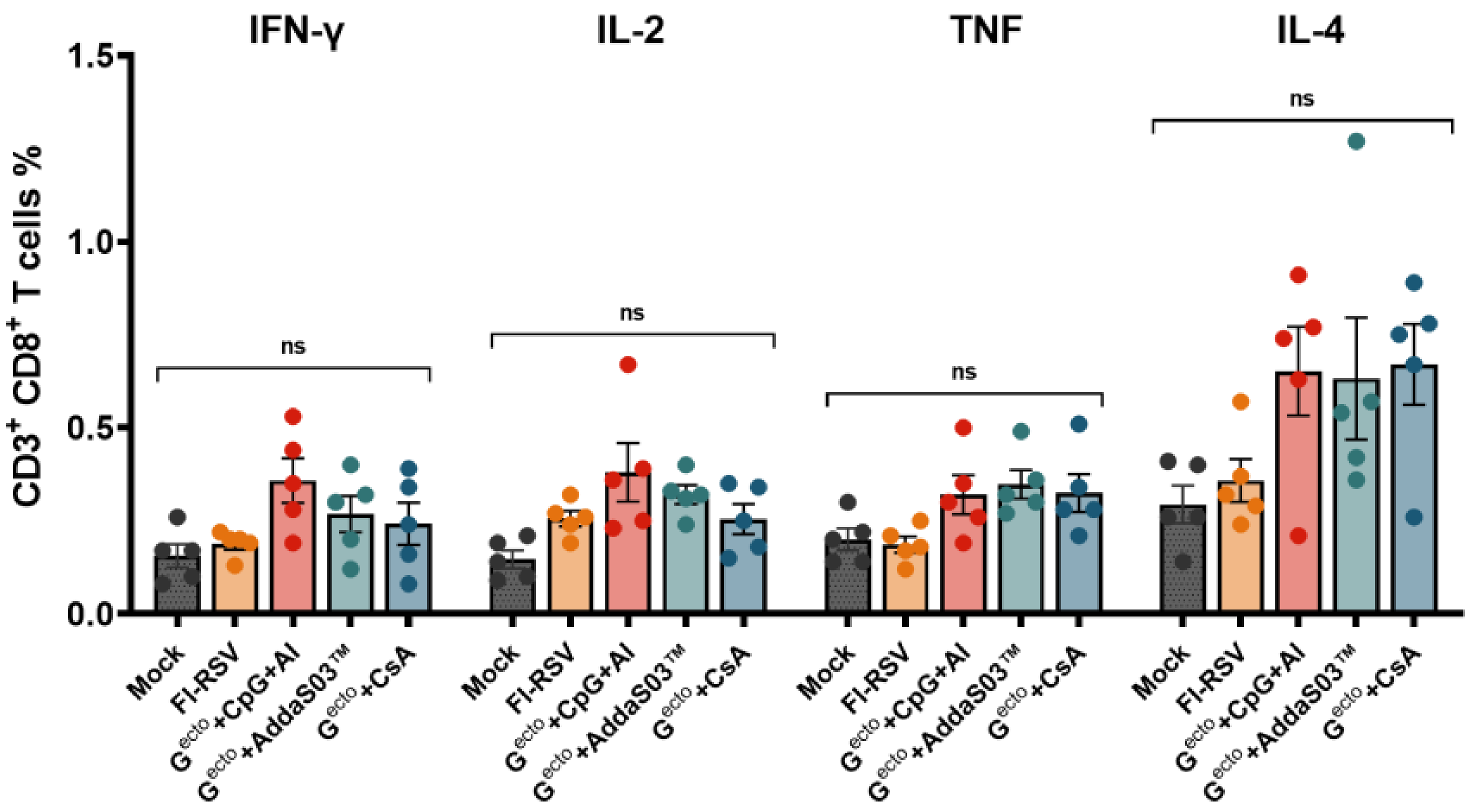

Supplementary Figure S1. Detection of intracellular cytokines IFN-γ, IL-2, TNF, and IL-4 in CD8+T cells from Gecto-immunized mice.

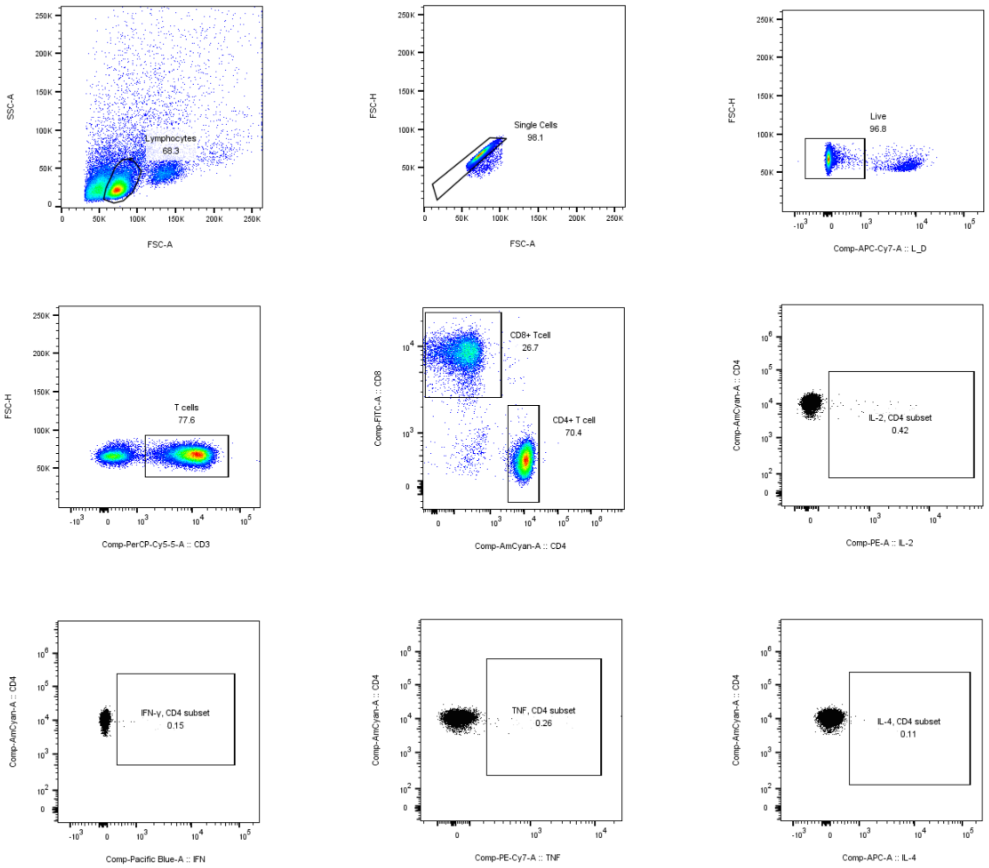

Supplementary Figure S2. Gating strategy for detection of CD4+ T cells intracellular cytokines. Exemplary gating strategy of Splenocytes samplefrom an G^{ecto}-immunized mouse. Splenocytes were collected 14 days later and the expression of IL-2, IFN-γ, TNF, and IL-4 in CD4+T cells.
